# In vitro bioaccessibility and antioxidant properties of edible bird’s nest following simulated human gastro-intestinal digestion

**DOI:** 10.1186/1472-6882-14-468

**Published:** 2014-12-05

**Authors:** Zhang Yida, Mustapha Umar Imam, Maznah Ismail

**Affiliations:** Laboratory of Molecular Biomedicine, Institute of Bioscience, Universiti Putra Malaysia, 43400 Serdang, Selangor Malaysia; Cardiology Department, Affiliated Hospital of Chengde Medical University, 067000 Chengde, Hebei China; Department of Nutrition and Dietetics, Faculty of Medicine and Health Sciences, Universiti Putra Malaysia, 43400 Serdang, Selangor Malaysia

**Keywords:** Antioxidant, Edible bird’s nest, Gastro-intestinal digestion

## Abstract

**Background:**

Edible birds’ nest (EBN) is reported to be antioxidant-rich. However, the fate of its antioxidants after oral consumption is not yet reported. To explore this, we hypothesized that EBN antioxidants are released from their matrix when subjected to in vitro simulated gastrointestinal digestion.

**Methods:**

EBN samples were extracted using hot water (100°C) with or without subsequent sequential enzymatic digestion using pepsin (10,000 units), pancreatin (36 mg) and bile extracts (112.5 mg). Additionally, pH changes (8.9 to 2 and back to 8.9) similar to the gut were applied, and a 10 KDa dialysis tubing was used to simulate gut absorption. The antioxidant capacities of the water extracts of EBN before and after digestion were then determined using ABTS and oxygen radical absorbance capacity (ORAC) assays, while the protective effects of the EBN samples against hydrogen peroxide-induced toxicity in HEPG2 cells were determined using MTT assay and acridine orange (AO)/propidium iodide (PI) staining.

**Results:**

Antioxidant assays (ABTS and ORAC) showed that the undigested EBN water extract had little antioxidant activity (1 and 1%, respectively at 1000 μg/mL) while at similar concentrations the digested samples had significantly (p < 0.05) enhanced antioxidant activities, for samples inside (38 and 50%, respectively at 1000 μg/mL) and outside (36 and 50%, respectively at 1000 μg/mL) the dialysis tubing, representing absorbed and unabsorbed samples, respectively. Cell viability and toxicity assays also suggested that the EBN extracts were non-toxic to HEPG2 cells (cell viabilities of over 80% at 1000 μg/mL), while AOPI showed that the extracts protected HEPG2 cells from hydrogen peroxide induced-toxicity.

**Conclusions:**

Based on the findings, it is likely that EBN bioactives are released from their matrix when digested in the gut and then absorbed through the gut by passive-mediated transport to exert their functional effects. However, there is need to confirm these findings using in vivo systems to determine their clinical significance.

## Background

Edible birds’ nest (EBN) has been used traditionally across much of Asia for its health promoting benefits. It is produced by swiftlet species, commonly found in Asian countries like Thailand, Indonesia and Malaysia. Its consumption among Asians has a long history and it is thought to improve overall general health [1, 2]. Interest in these claims has grown considerably over the years and studies have been conducted to demonstrate the effectiveness of EBN towards the claimed benefits. It is now known that EBN has antioxidant, anti-inflammatory, and bone strengthening properties among others [2–4]. EBN contains many bioactive compounds that are thought to be responsible for its health promoting effects including glucosamine, lactoferrin, sialic acid, amino acids, fatty acids, triacylglycerol, vitamins, minerals and other antioxidants [1, 2, 5–7].

Antioxidants have received close attention in recent years because of their perceived efficacy towards relieving oxidative stress-related diseases, which are thought to be growing in incidence globally. In fact, most chronic diseases have been linked to oxidative stress and studies have demonstrated that the use of antioxidants could play significant roles in reducing risk and in managing the diseases [8]. However, high antioxidant composition does not always equate to better efficacy since nutrikinetic factors could determine the bioavailability of bioactives from food sources and hence their bioactivity [9].

The use of in vitro systems that simulate gastrointestinal digestion has been shown to provide insights into the amounts of bioactives that are likely to be derived from foods when they are consumed. In this regard, foods are subjected to conditions similar to what obtains in the gut and the results indicate whether such conditions would lead to release of antioxidants present in the foods or not [10]. Thus, in this study we demonstrate the effect of simulated gastrointestinal digestion on antioxidant properties of EBN to provide insights on the degree to which antioxidants in EBN are made available after it undergoes digestion.

## Methods

### Reagents

Hydrogen peroxide (H_2_O_2_) was purchased from Bendosen Laboratory Chemicals (Selangor, Malaysia). Pepsin, pancreatin, bile extract, sodium biocarbonate (NaHCO_3_), fluorescein sodium salt, 2,2′-azobis (2-amidinopropane) dihydrochloride (AAPH), dichloro-dihydro-fluorescein diacetate (DCFH-DA), RPMI 1640 medium, fetal bovine serum, antibiotic, potassium persulphate (K_2_S_2_O_8_), 2,2′-azino-bis[3-ethylbenzothiazoline-6-sulphonic acid] (ABTS) reagent, and 3-(4,5-Dimethylthiazol-2-yl)-2,5-diphenyltetrazolium bromide (MTT) powder were purchased from Sigma-Aldrich (St. Louis, MO, USA), while other cell culture materials were purchased from BD Biosciences (NJ, USA).

### EBN sample preparation

EBN was supplied by Blossom View Sdn. Bhd (Terrengganu, Malaysia). Upon collection, it was cleaned under tap water for 5 mins, dried at room temperature and ground into powder manually using mortar and pestle. The ground EBN (1 g) was dissolved in 100 mL distilled water, incubated at 37°C for 2 h and boiled (100°C) for 30 min afterwards. The rest of the EBN was stored at -80°C until further analysis, while the boiled sample was kept in a shaking incubator (LSI-3016, Daihan Lab tech Co. Ltd, Korea) at 55°C and 50 rpm for 3 h. At this point, the pH (Mettler Toledo, MP 125, Switzerland) of the sample was determined to be 8.9.

### Simutated gastro-intestinal digestion

The simulated digestion was done as reported by Gil-Izquierdo, Zafrilla and Tomás-Barberán [11], with minor modifications. Briefly, to simulate gastric digestion, 1 g EBN sample was dissolved in 100 mL distilled water, and kept at 37°C for 2 h in a shaking incubator (50 rpm). The mixture was then boiled (100°C) for another 30 min and kept at 55°C for 3 h on the shaking incubator (50 rpm). The pH of 8.9 was adjusted to 2.0 using HCL, and pepsin (10,000 units) was added. The pH-adjusted sample was subjected to shaking for another 2 h, and boiling for 20 min (to inactivate pepsin) before bringing it in contact with a dialysis tubing. Snakeskin Pleated Dialysis Tubing (10 K MWCO, Thermo Fisher Scientific Inc., Waltham, USA) containing 2 M NaHCO_3_ was immersed into the container holding the pepsin-digested EBN samples and subjected to continuous shaking for 10 min until the pH was 8.0. Pancreatin digestion (36 mg) in the presence of bile extract (112.5 mg) was then carried out on the sample with continuous shaking for 2 h to simulate small intestinal conditions. The sample was boiled after this for 30 min and cooled, at which point the pH was 8.9. The water samples within and outside the dialysis tubing in the container holding the sample was collected and filtered (0.45 μm pore size, Fisher Scientific, Santa Clara, CA, USA). The filtered sample was then used for analyses.

### In vitro antixodant testing

#### ABTS and ORAC assays

Antioxidant potentials of the EBN samples before and after the simulated gastro-intestinal digestion were analysed by investigating their abilities to scavenge the ABTS free radical using the method described previously [12]. Briefly, K_2_S_2_O_8_ solution (2.45 uM) was prepared freshly by dissolving 6.62 mg of K_2_S_2_O_8_ in 10 mL of distilled water, while 7 mM ABTS was prepared by dissolving 38.4 mg in 10 mL distilled water. The reagents (K_2_S_2_O_8_ and ABTS) were mixed and incubated in the dark at room temperature for 16 h prior to use. The spectrophotometric absorbance of the mixture at 735 nm was determined to be 0.700 ± 0.005 before use. Then, 20 uL EBN (4-1000 μg/mL) sample or Trolox standard (1.56-100 μg/mL) was mixed with 200 uL of the diluted ABTS solution in a 96 well plate, and the absorbance read at 734 nm. The ABTS radical cation scavenging activity was calculated as the percentage reduction in absorbance, represented by the equation (y = 1.71761x + 1.3953, R^2^ = 0.9939).

For ORAC [13], trolox standards (1.56-100 μg/mL) and EBN samples (4-1000 μg/mL) were used. Briefly, 150 uL 8.16 × 10^−5^ mM fluorescein was added to each well of 96 well plate and 25 uL sample or standard was added. The mixture was then incubated at 37°C for 15 mins, and 25 uL AAPH solvent (153 uM) was added. The fluorescence data was measured using BioTeK Synergy H1 Hybrid Reader (BioTek Instruments Inc., Winooski, VT, USA) with the excitation wave length of 485 nm and emission wave length of 520 nm. The plate was read continuously at intervals of one minute for another 2 h at 37°C. ORAC levels were expressed as mole of Trolox equivalents (TE) per mole of antioxidant standard.

### Cell culture

HEPG2 cell line was acquired from ATCC, and cultured using RPMI medium containing glucose, FBS and penicillin/streptomycin. The cell culture was maintained in a humidified incubator at 37°C under 5% CO_2_. Upon 80% confluence in a 75 cm^3^ flask, cells were seeded into 96 well plate at a concentration of 1*10^5^ and incubated for 24 h at 37°C. Then EBN samples (water-extracted and digested, 1.95-1000 ppm) were used to treat the cells for 24 h. MTT was later added to the wells and incubated for another 4 h at 37°C in the dark. Absorbance was read using BioTeK Synergy H1 Hybrid Reader (BioTek Instruments Inc., Winooski, VT, USA) at 540 nm, to detect the amount of MTT formazan product formed.

In another experiment, 300 uM H_2_O_2_ (determined as IC50 from preliminary experiment) was added to HEPG2 cells seeded on a 96 well plate to induce stress, subsequent to treatment with the EBN samples. H_2_O_2_-treated cells were incubated for 1 h, and MTT added for another 4 h as stated above. Absorbance was read at 540 nm and cell viability was calculated by using following equation:


### Intracellular antioxidant (DCFH-DA) assay

DCFH-DA assay was done as reported previously, with minor modifications [14]. Briefly, HEPG2 cells were plated at a density of 1 × 10^5^/well into 96-well plate and allowed to attach for 24 h. Cells were then washed with PBS and incubated with 10 μM DCFH-DA in the medium under 5% CO_2_/95% air at 37°C for 30 min. Then, cells were washed again using PBS and incubated with the different treatments (4-1000 ppm EBN) for 24 h. H2O2 (300 μM) was then added to the cells and the plate was placed on the BioTeK Synergy H1 Hybrid Reader (BioTek Instruments Inc., Winooski, VT, USA) with temperature maintained at 37°C. The excitation filter was set at 480 nm and the emission filter was set at 510 nm. The fluorescence from each well was captured continuously and the data points were recorded every 10 min. The data were exported to Excel (Microsoft, Seattle, WA, USA) spreadsheet software and used to plot a line graph showing changes in ROS generation over time.

### Acridine Orange and Propidium Iodide (AO/PI) staining using fluorescence microscope

HepG2 cells were seeded, and later treated with EBN and 300 μM H_2_O_2_ as previously described [15]. After the incubation period, the growth media was discarded and the cells stained with the dye mixture (10 μL of 1 mg/mL AO and 10 μL of 1 mg/mL PI). Stained cells were examined using a confocal microscope (Olympus, Tokyo, Japan). Multiple independent images were taken.

### Statistical analysis

All data are presented as mean ± SD. The data was evaluated by one-way ANOVA using Statistical Package for Social Sciences software, version 20 (SPSS Inc., Chicago, IL). Differences between the means were assessed using Tukey’s multiple comparisons and Student’s *t*-test. Statistical significance was considered at *p* < 0.05.

## Results and discussion

### Antioxidant potentials of EBN samples

**Figure 1:**
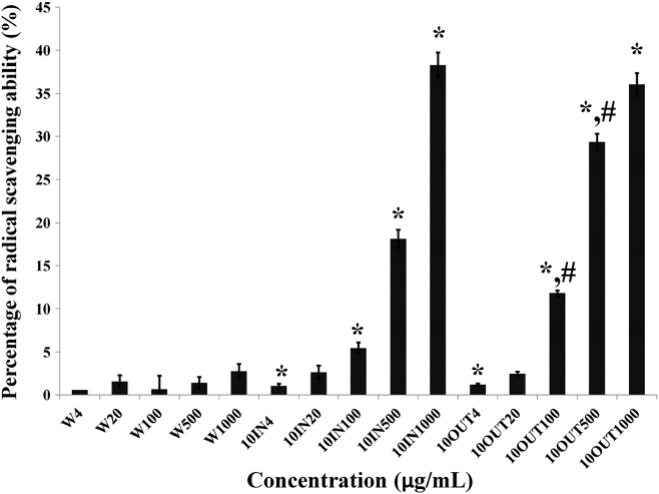
**ABTS results showing the antioxidant potentials of edible birds’ nest (EBN) before and after simulated gastrointestinal digestion.** Water extracts (W), digested samples absorbed into tube (10IN), and digested samples remaining outside the tube (10OUT) showed dose dependent (4-1000 μg/mL) antioxidant potential. *indicates significant difference (p < 0.05) in comparison with corresponding concentration of W. ^#^indicates significant difference (p < 0.05) in comparison with corresponding concentration of 10IN.

Figure 1 shows the ABTS scavenging potentials of EBN samples. Water extract of EBN samples, over the range of 4 to 1000 μg/mL, prior to digestion had the lowest scavenging potential (with a maximum of 1%) compared to the samples subjected to simulated gastrointestinal digestion. In comparison, simulated gastrointestinal digestion increased the scavenging potential (p < 0.05) due to EBN with slightly higher values for samples remaining outside the dialysis tubing (38% at 1000 μg/mL) compared with samples inside the tubing (36% at 1000 μg/mL). The dialysis tubing represents a model of absorption in the gastrointestinal system, and the results suggested that following consumption of EBN, it is likely that the unabsorbed antioxidants would be higher than those absorbed. This is in line with what has been reported previously [16]; lactoferrin is thought to be absorbed minimally with most of it remaining in the gut where it modulates gut health, and the same may be true for other bioactives. The findings from this experiment also suggest that digestion of EBN leads to release of it bioactive compounds, in line with what is expected of food bioactives under gastrointestinal digestion [9].

**Figure 2:**
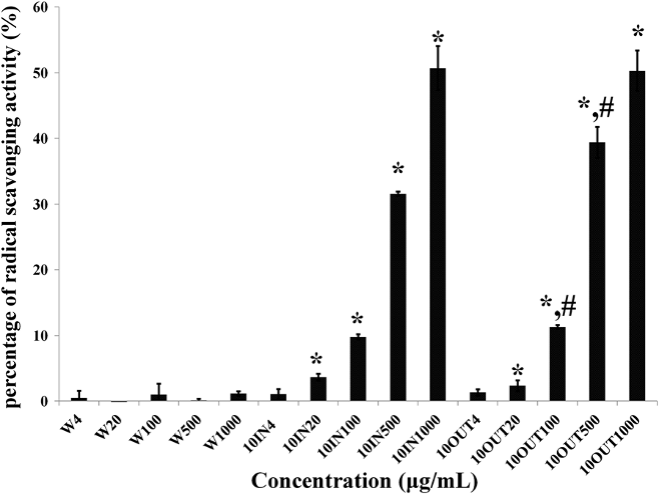
**Oxygen radical absorbance capactiy of edible birds’ nest (EBN) before and after simulated gastrointestinal digestion.** Water extracts (W), digested samples absorbed into tube (10IN), and digested samples remaining outside the tube (10OUT) showed dose dependent (4-1000 μg/mL) antioxidant potential. *indicates significant difference (p < 0.05) in comparison with corresponding concentration of W. ^#^indicates significant difference (p < 0.05) in comparison with corresponding concentration of 10IN.

Furthermore, ORAC results on Figure 2 show similar patterns of scavenging activities to the ABTS results. Water extract of EBN showed little scavenging activity (1%) even at the highest concentration used, compared with the samples subjected to simulated gastrointestinal digestion, which showed a dose-dependent activity that was maximal (50%) for samples in and outside the tubing at the highest concentration (1000 μg/mL). The similar scavenging activities observed for samples within and outside the dialysis tubing, suggested that as much antioxidants absorbed are left in the gut likely through passive-mediated transport of the molecules responsible for the antioxidant activity. These results suggest that when EBN is digested in the gut, much of its antioxidants are absorbed through the gut while others remain within the lumen to exact local effects [16]. Also, the ORAC results indicate that these findings may have clinical significance since ORAC has been reported to reproduce what happens in biological systems.

### Cell toxicity and viability assays

**Figure 3:**
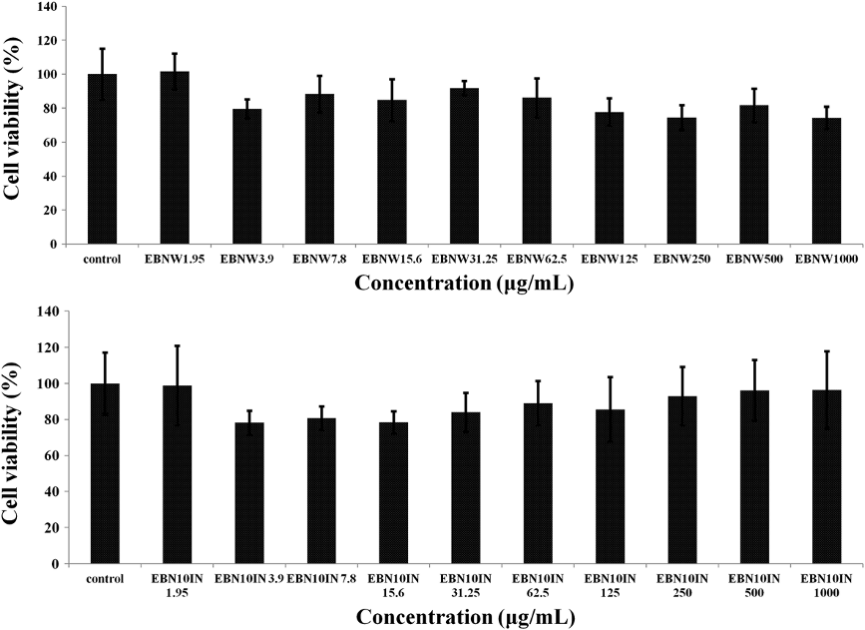
**Cytotoxic effects of a) water extract and b) digested samples of edible birds’ nest (EBN) on HepG2 cells.** EBN water extract (EBNW) and digested samples (EBN10IN) showed little toxicity to HepG2 cells over concentrations of 1.95 and 1000 μg/mL. No significant differences were observed (p > 0.05) between the control and different concentrations of EBNW or EBN10IN.

**Figure 4:**
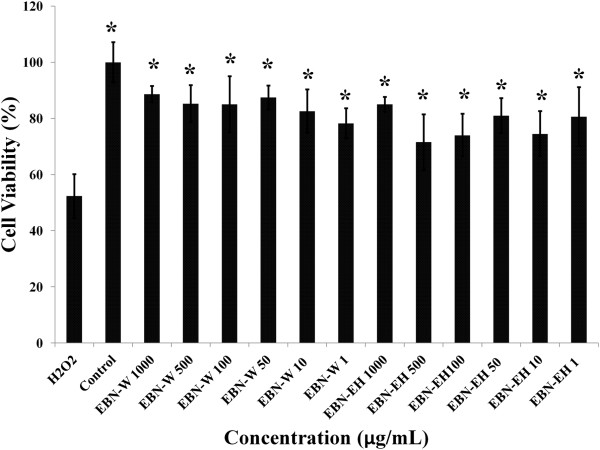
**Cytoprotective effects of edible birds’ nest (EBN, 1-1000 μg/mL) against H**_**2**_ **O**_**2**_ **in HepG2 cells.** EBN water extract (EBNW) and digested samples (EBN10IN) protected HepG2 cells from toxic effects of H_2_O_2_. *significantly higher than H_2_O_2_ (p < 0.05).

To determine the effects of the EBN water extracts with and without digestion, the MTT assay was used. After treatment for 24 h, there were no significant signs of toxicity due any of the EBN extracts. Figure 3 shows the cytotoxicity results for water extracts of EBN before and after digestion (sample within dialysis tubing), respectively. The non-digested samples showed a cell viability of more than 80% over the entire range of treatments. The digested samples also showed similar results but interestingly had higher cell viabilities with higher concentrations of the extract. Additionally, as can be recalled, 300 uM H_2_O_2_ produced 50% cell viability on HEPG2 cells but in the presence of the EBN extracts (digested and undigested), cell viability was over 80% across all tested doses (Figure 4).

### Intracellular antioxidant assay (DCFH-DA) assay

**Figure 5:**
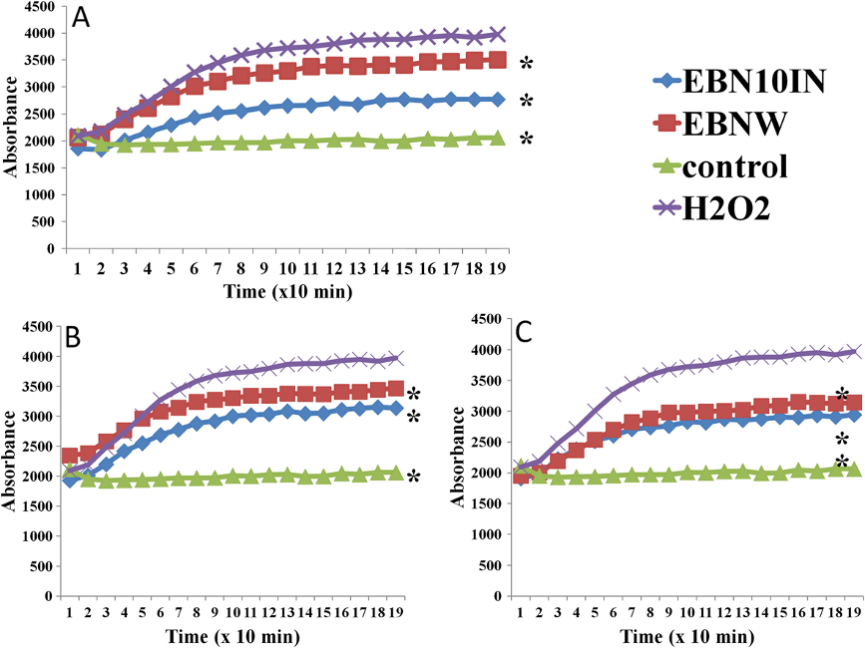
**Effect of edible birds’ nest (EBN) (A) 4, (B) 20 and (C) 500 μg/mL on intracellular ROS determination using DCFH-DA.** Water extracts (EBNW) and digested samples (EBNW10IN), reduced levels of ROS generation compared with the H_2_O_2_-treated group. *significantly lower than H_2_O_2_ (p < 0.05).

The DCFH-DA assay is used to measure intracellular antioxidant activity and although it has limitations, it has been shown to provide valuable insights into possible antioxidant activity [14]. In the present study, the addition of H_2_O_2_ showed high absorbance readings (fluorescence unit of 3975 after 190 mins, Figure 5A) indicating that high numbers of the cells had died. On the other hand, in the presence of the EBN extracts (4, 20 and 500 μg/mL, Figures 5A, B and C, respectively), there was a reduction of this intensity especially with the digested samples (2700, 3100 and 2900 fluorescence units, respectively), which tended towards normal (fluorescence unit of 2000). These results also suggest that in the presence of the EBN extracts, HEPG2 cells are conferred some protection against H_2_O_2_.

### AOPI staining

**Figure 6:**
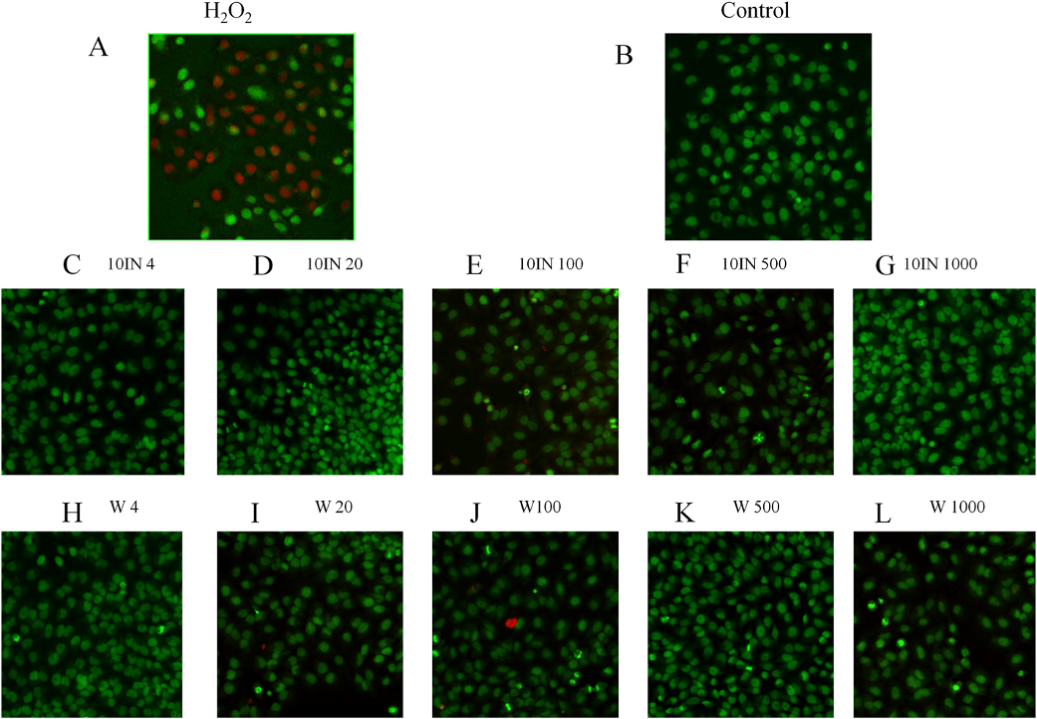
**AOPI staining of HepG2 cells after treatment with edible birds’ nest (EBN) and H**_**2**_ **O**_**2**_ **.** **(A)** H_2_O_2_-treated, **(B)** untreated control, **(C-G)** 4-1000 μg/mL digested samples (10IN), **(H-L)** 4-1000 μg/mL water extracts. H_2_O_2_ showed signs of cell death (red colored cells), while EBN protected cells from death (live cells colored green).

When cells are stained with AOPI, the dead cells produce a classical orange to red flourescence while live cells will show a green glow [15]. In this study, H_2_O_2_ showed a pattern indicative of dead cells with AOPI staining (Figure 6A) in contrast to untreated controls that showed no signs of dead cells (Figure 6B). Treatment with extracts in the presence of H_2_O_2_ protected the cells from death as shown on Figures 6C-6L.

In aggregate, the findings in this study are in agreement that EBN water extract produces antioxidant activity which is more pronounced when the samples are digested, as suggested by the results of the simulated gastrointestinal digestion in this study. The findings also suggest that almost an equal amount of antioxidants are likely absorbed by the gut as those unabsorbed following digestion of EBN. These indicate that the antioxidants bound to their matrix in EBN are released upon digestion and will hence be available for bioactivity, as shown by the results of enhanced antioxidant activity in the ORAC assay for the digested samples that collected in and out of the dialysis tubing in comparison to the undigested water extract. The results of the cell toxicity and viability assays support this hypothesis, which was further corroborated by the AOPI staining results.

## Conclusions

In this study, EBN samples subjected to simulated gut digestion showed enhanced antioxidant activity even in the presence of H_2_O_2_ as evidenced by the results of antioxidant, and cell viability and cytotoxicity assays. The findings were further supported by the results of AOPI staining. The overall results suggest that when EBN is subjected to gut digestion, its antioxidants are released from their bound matrix and may exert significant antioxidant activity. The findings show that EBN has enhanced antioxidant activity after digestion, which may possibly underlie some of its functional effects after consumption, and is worth studying further.

## Abbreviations

AAPH: 2,2′-azobis (2-amidinopropane) dihydrochloride
ABTS: 2,2′-azino-bis[3-ethylbenzothiazoline-6-sulphonic acid
AO-PI: Acridine orange–propidium iodide
DCFH-DA: Dichloro-dihydro-fluorescein diacetate
EBN: Edible birds’ nest
H_2_O_2_: hydrogen peroxide
K_2_S_2_O_8_: Potassium persulphate
MTT: (3-[4,5-dimethylthiazol-2-yl]-2,5-diphenyl-tetrazolium bromide)
NaHCO_3_: Sodium biocarbonate
ROS: Reactive oxygen species.
